# Supplementary material for: Global burden and epidemiological prediction of polycystic ovary syndrome from 1990 to 2019: A systematic analysis from the Global Burden of Disease Study 2019
Source: PLoS One. 2024 Jul 18;19(7):e0306991. doi: 10.1371/journal.pone.0306991 (PMC11257291; doi:10.1371/journal.pone.0306991)
Supplement: S1 File — (PDF) [file pone.0306991.s001.pdf]

Supplementary Materials

S1 Fig. Packages used in data processing, analysis and prediction.

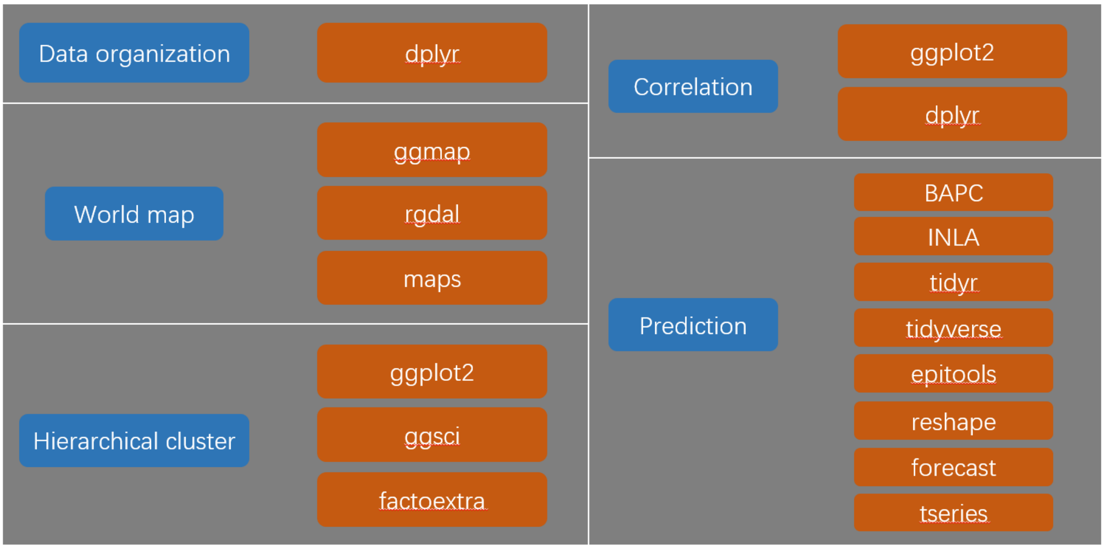

S2 Fig. National burden of PCOS. (A) estimates of ASPR in 2019; (B) national EAPC of prevalence.

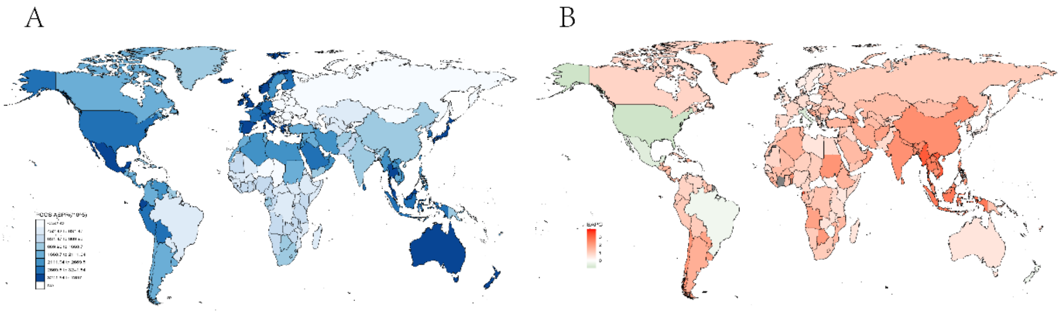

S3 Fig: ARIMA prediction of PCOS burden in the next 20 years. (A) Time series of ASPR and ASIR after 3 times of difference. (B) ACF and PACF of ASPR and ASIR. (C) Global ASPR and ASIR from 1990 to 2042.

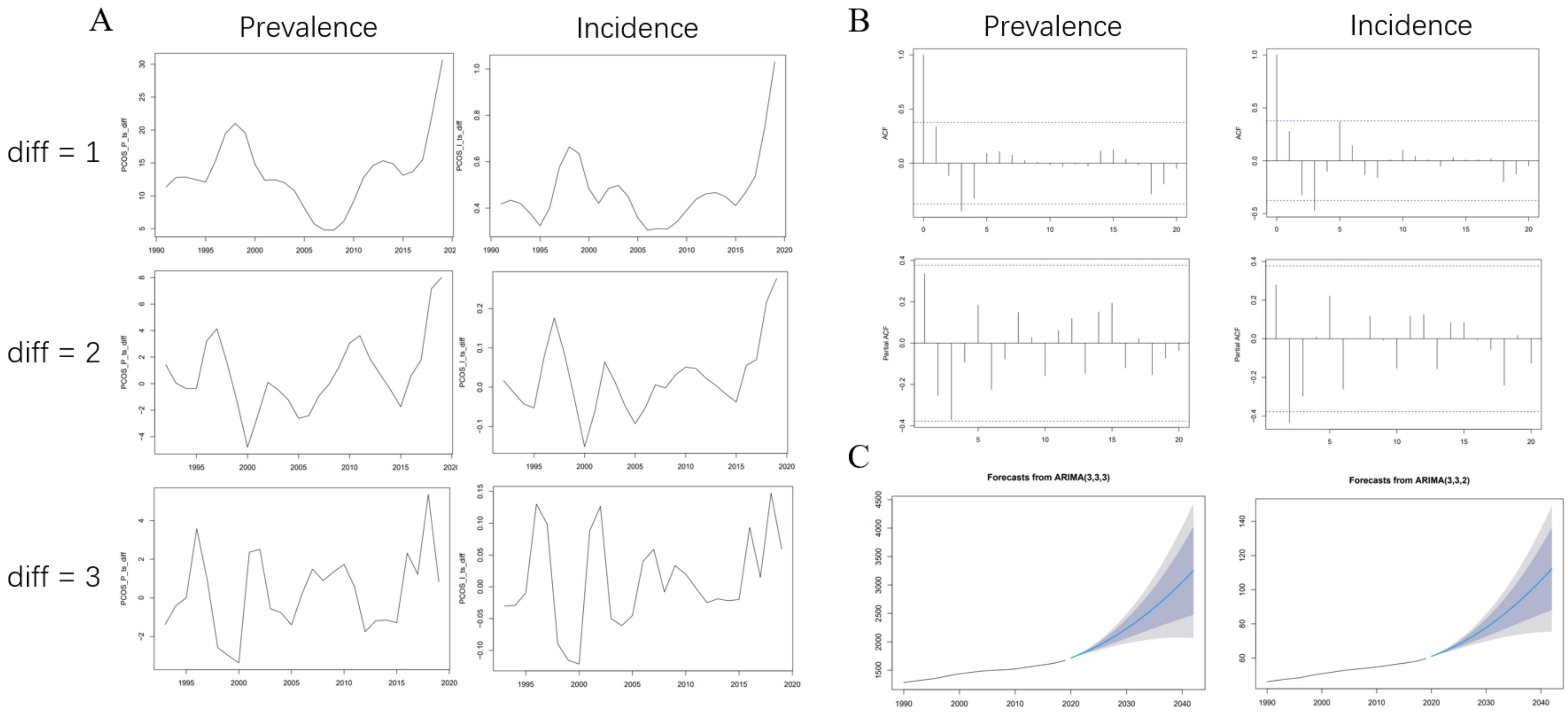

S1 Table. Analysis of incidence and prevalence in age-region groups from 1990 to 2019.

Regionns	Years	Measures	10 to 14	15 to 19	20 to 24	25 to 29	30 to 34	35 to 39	40 to 44	45 to 49	50 to 54
Global	1990	prevalence	1003144	4520510	5994914	5679853	5039306	4590067	3974250	2781041	679988.8
Global	1990	incidence	593661.7	705696.9	37919.93	19543.18	9436.171	6451.639	3361.154	1442.788	411.1557
Global	2019	prevalence	1653030	7204381	9763356	1025159 0	1021406 4	9330481	8534464	7303397	1737555
Global	2019	incidence	978325.4	1038396	46065.52	27190.1	15347.2	10142.31	6041.029	3095.674	908.2229
African Region	1990	prevalence	46984.52	210024.1	247531.2	214532.9	172227.9	136029.8	101436.7	72184.84	18822.93
African Region	1990	incidence	27827.95	32539.99	3862.104	1868.366	837.3069	494.2503	241.0538	106.6958	27.05532
African Region	2019	prevalence	135072.3	611358.8	717202.4	657176.1	576229.2	480983.9	382673.6	274149.8	61345.02
African Region	2019	incidence	79996.06	91609.52	8890.701	4377.693	2068.74	1276.83	667.9301	282.4905	70.32213
Eastern Mediterranean Region	1990	prevalence	102753.3	389043.4	420225.6	351879.8	287784.8	236628.7	185082.5	125113.4	27852.64
Eastern Mediterranean Region	1990	incidence	60838.09	48496.92	2359.847	1150.234	553.6685	351.0726	188.6618	83.96495	21.74787
Eastern Mediterranean Region	2019	prevalence	210284.4	828544.8	1000553	965458.8	939964.1	845381.3	667058.2	461434.6	94823.13
Eastern Mediterranean Region	2019	incidence	124490.9	100063.1	4362.641	2401.604	1336.045	907.1823	493.7429	217.2125	54.31558
European Region	1990	prevalence	208574	848285.3	1150076	1183210	1086093	1004124	957000.5	703883.3	181329.9
European Region	1990	incidence	123332.1	101775.1	3114.855	1985.236	1150.777	804.0912	466.2549	205.9748	76.67914
European Region	2019	prevalence	222949.4	822697.4	1123641	1251062	1303074	1318638	1319818	1170766	283651.6
European Region	2019	incidence	131868.9	101779.4	2718.989	1861.527	1231.604	911.914	580.361	300.1461	94.8049
Region of the	1990	prevalence	244118.3	953820	1300633	1303996	1216357	1043750	860645.4	566582.6	140421.7

**Americas**											
**Region of the Americas**	1990	incidence	144474.2	147290.4	3889.493	2180.046	1122.173	730.7863	401.821	169.2135	44.72391
**Region of the Americas**	2019	prevalence	306195.1	1290537	1849411	1872339	1755414	1648456	1465796	1181310	322167.5
**Region of the Americas**	2019	incidence	181202.8	203732.5	4583.501	2696.139	1503.738	1093.65	656.4587	323.7741	96.47053
**South-East Asia Region**	1990	prevalence	178328.4	760847.2	955157	874751.1	742403.6	616576.9	485586.2	344681.2	83631.02
**South-East Asia Region**	1990	incidence	105525.8	110908.1	10215.82	5290.568	2551.734	1640.074	861.5895	380.3142	98.27096
**South-East Asia Region**	2019	prevalence	448515.9	2022290	2627658	2536768	2410133	2243729	1962382	1501741	337051.8
**South-East Asia Region**	2019	incidence	265404.9	267316.9	15711.44	8480.839	4496.107	3106.142	1789.974	835.64	224.9215
**Western Pacific Region**	1990	prevalence	218843.6	1343475	1903087	1732477	1517306	1538951	1372792	960748.1	225925.6
**Western Pacific Region**	1990	incidence	129566.6	262872.3	14381.89	7006.386	3187.959	2411.885	1191.274	492.5889	141.4716
**Western Pacific Region**	2019	prevalence	324363.3	1606066	2413968	2936392	3197796	2762444	2708909	2689938	632681.6
**Western Pacific Region**	2019	incidence	192017.5	271236.7	9691.671	7302.45	4669.504	2814.971	1833.673	1126.636	364.3158
**High SDI**	1990	prevalence	234317.9	1267736	1975646	2060079	1974589	1891754	1829098	1265632	288603.7
**High SDI**	1990	incidence	138682.2	267721.4	3446.337	2098.738	1141.017	810.1903	508.068	222.1841	66.42755
**High SDI**	2019	prevalence	262841.4	1323844	2143094	2322005	2361842	2388336	2269136	2016982	464565.2
**High SDI**	2019	incidence	155556.4	278255.4	3156.89	2006.334	1189.01	901.7118	558.5973	307.2202	98.70876
**High-middle SDI**	1990	prevalence	123569	118652.4	7477.833	4171.567	2173.086	1521.899	769.4478	316.5742	104.4692

**High-middle SDI**	1990	incidence	208937.9	919457.2	1194495	1176100	1067719	975631.2	826321.5	587008.2	158637.2
**High-middle SDI**	2019	prevalence	155643.9	141089.3	6281.691	4555.521	3005.755	1988.349	1244.235	677.7396	210.6481
**High-middle SDI**	2019	incidence	263086.3	1097381	1538151	1830873	2016540	1830845	1772320	1584590	384567.6
**Middle SDI**	1990	prevalence	397885.3	1634884	1996179	1720457	1400579	1209082	918144.4	644878.5	158863
**Middle SDI**	1990	incidence	235463.7	215468.9	15202.25	7435.784	3374.858	2326.729	1151.719	487.8736	132.5386
**Middle SDI**	2019	prevalence	690545.4	2806883	3690982	3905442	3875703	3404640	3045741	2619898	635372.6
**Middle SDI**	2019	incidence	408601.5	343800	14844.64	9475.494	5529.671	3579.59	2176.694	1172.72	354.9439
**Low-middle SDI**	1990	prevalence	121979.6	524056.1	625846.8	542953.1	447406.8	386424.5	303214.7	214157.4	56055.03
**Low-middle SDI**	1990	incidence	72240.29	76380.32	8245.463	4070.624	1920.513	1267.332	667.5364	297.8821	77.62707
**Low-middle SDI**	2019	prevalence	301152	1369437	1708060	1604838	1458911	1282728	1110103	846408.9	201711.8
**Low-middle SDI**	2019	incidence	178326.7	185000.4	13354.69	7089.833	3660.173	2420.118	1398.292	658.6338	176.5013
**Low SDI**	1990	prevalence	39319.09	171320.1	199012.4	176780	146089.1	124679	95330.93	67813.39	17464.18
**Low SDI**	1990	incidence	23289.74	27080.67	3530.62	1757.164	822.2433	522.675	262.8476	117.5901	29.90486
**Low SDI**	2019	prevalence	134083.8	601428.1	676193.8	581621.6	494579.2	417932.7	331756.2	231074.8	50313.89
**Low SDI**	2019	incidence	79414.94	89583.38	8404.29	4049.507	1955.172	1247.425	660.1945	277.8895	66.99993
**Commonwealth High Income**	1990	prevalence	33703.89	158987.4	246615.3	260139.2	236706.8	213014.9	209625.9	143605.8	30181.83
**Commonwealth High Income**	1990	incidence	19946.2	26483.82	464.199	284.8863	149.0997	103.7208	69.91702	29.85563	8.462919
**Commonwealth High Income**	2019	prevalence	49472.7	193642.4	309906	357131.5	356738.5	341437	303830.4	268674.9	65688.09
**Commonwealth High Income**	2019	incidence	29271.74	33046.12	463.8885	306.4541	178.8043	132.0205	79.25734	44.28482	14.72618
**Commonwealth Middle Income**	1990	prevalence	141045.1	568208.6	700074.2	639500.8	542301.7	460488.4	366221	244786.1	64768.66
**Commonwealth Middle Income**	1990	incidence	83446.64	80187.02	9438.442	4787.941	2287.254	1485.096	782.5288	344.1628	87.03892
**Commonwealth**	2019	prevalence	370988.8	1688758	2148693	2066864	1920445	1692667	1397668	1002423	239655.5

**Middle Income**											
**Commonwealth Middle Income**	2019	incidence	219530	225021.2	17539.98	9166.81	4671.887	3099.214	1721.575	785.1016	205.5837
**Commonwealth Low Income**	1990	prevalence	14911.29	69024.39	80078.96	68318.36	53386.27	41528.19	31372.54	21377.65	5396.485
**Commonwealth Low Income**	1990	incidence	8833.158	10911.99	1303.156	627.6533	280.72	164.3398	83.07294	35.2508	8.842414
**Commonwealth Low Income**	2019	prevalence	39830.29	194887.1	227752.7	203503.4	175602.5	146382.5	121375	83948.1	18858.03
**Commonwealth Low Income**	2019	incidence	23593.43	28751.18	2623.704	1331.012	655.6981	412.4579	227.8696	98.42609	24.29252

S2 Table. Analysis of incidence and prevalence in time series-region groups from 1990 to 2019.

Years	global		African Region		Region of the Americas		South-East Asia Region		European Region		Eastern Mediterranean Region		Western Pacific Region		High SDI		High-middle SDI		Middle SDI		Low-middle SDI		Low SDI		Commonwealth High Income		Commonwealth Middle Income		Commonwealth Low Income	
	prevalence	incidence	prevalence	incidence	prevalence	incidence	prevalence	incidence	prevalence	incidence	prevalence	incidence	prevalence	incidence	prevalence	incidence	prevalence	incidence	prevalence	incidence	prevalence	incidence	prevalence	incidence	prevalence	incidence	prevalence	incidence	prevalence	incidence
**1990**	1286.222	46.13567	525.0086	20.64294	2044.324	77.33861	794.6117	30.69365	1709.161	62.88532	1257.078	47.75284	1312.339	47.8611	2990.398	121.2578	1188.805	45.62611	1132.666	46.46044	603.0921	23.94843	444.5317	17.89007	2651.907	107.34	672.4884	26.30448	406.9257	16.32195
**1991**	1297.604	46.55351	533.5037	20.91175	2085.626	78.94229	805.4618	31.02302	1732.489	62.85756	1260.932	47.8473	1312.813	48.65929	3023.096	122.9779	1199.252	45.71195	1153.723	47.56505	611.4982	24.26586	447.3363	17.96851	2723.298	110.1773	679.5312	26.54623	410.0341	16.44215
**1**	13	4	54	2	21	8	81	3	17	6	12	4	13	4	30	1	12	4	11	4	62	2	45	1	27	1	68	2	41	1

**992**	10.406	6.98761	2.5478	1.19898	22.538	0.42916	7.7482	1.38516	54.543	2.8623	65.157	7.99578	18.989	9.54753	54.848	24.358	12.447	5.88306	77.455	8.67797	0.7594	4.60485	0.7499	8.06352	91.38	12.8541	7.8416	6.81888	3.0874	6.56249
**1993**	1323.252	47.40784	551.6311	21.49446	2153.477	81.74461	830.9821	31.76286	1771.492	62.8349	1271.935	48.26591	1329.379	50.4559	3085.906	125.3782	1225.714	46.09182	1202.496	49.75654	630.44	24.95036	453.8267	18.15714	2855.968	115.3472	697.0636	27.11375	415.9127	16.67845
**1994**	1335.726	47.78499	560.59	21.79114	2178.153	82.79477	844.7865	32.14466	1785.094	62.78629	1281.64	48.6454	1343.205	51.34982	3114.563	126.0632	1239.341	46.31528	1227.972	50.74542	640.1856	25.29045	456.7555	18.26156	2916.033	117.6078	706.9205	27.42672	418.9044	16.80722
**1995**	1347.831	48.10943	569.4338	22.08457	2195.175	83.50532	858.8117	32.52209	1797.08	62.76941	1293.029	49.09577	1361.372	52.17554	3140.497	126.4626	1253.712	46.55553	1252.797	51.58112	649.6015	25.61615	459.9964	18.38427	2970.941	119.6875	717.1264	27.75193	421.9055	16.93975
**19**	1363	48.	580.	22.	2207	84.	874.	32.	1811	62.	1307	49.	1389	53.	3170	12	1273	46.	1281	52.	660.	25.	464.	18.	3026	12	728.	28.	425.	17.

**96**	.147	51129	4069	45762	.852	09816	2698	94577	.096	82184	.912	69061	.514	12073	.424	7.0663	.65	96057	.236	40081	2314	98564	4318	56404	.344	1.9372	9675	13806	5442	09946
**1997**	1382.6	49.08924	594.4455	22.9427	2218.043	84.70636	892.2485	33.44738	1828.694	63.03211	1326.922	50.45081	1427.688	54.4361	3204.609	128.1877	1302.137	47.66546	1315.85	53.34493	673.0602	26.43335	470.3715	18.81554	3083.577	124.3718	743.2758	28.61018	430.2104	17.30163
**1998**	1403.613	49.75328	609.2974	23.45237	2225.136	85.26789	911.6445	33.99008	1845.769	63.44888	1348.311	51.28233	1470.712	55.88965	3238.526	129.5194	1333.667	48.58476	1352.756	54.33718	686.8996	26.92289	476.9264	19.09657	3136.903	126.7115	758.5666	29.11166	435.3255	17.52106
**1999**	1423.199	50.38778	622.5831	23.89812	2228.147	85.69857	931.4575	34.53675	1860.627	63.98548	1370.462	52.07686	1512.657	57.20582	3265.447	130.7236	1364.138	49.58404	1388.593	55.3049	700.5892	27.41716	483.3001	19.36851	3180.455	128.6035	773.5475	29.58585	440.3527	17.73662
**200**	1437.9	50.8	631.99	24.1	2225.0	85.8	950.79	35.0	1870.9	64.5	1390.6	52.7	1546.2	58.0	3279.4	131.	1389.8	50.5	1419.1	56.1	713.17	27.8	488.87	19.5	3207.9	129.	787.10	29.9	444.65	17.9

**0**	95	7124	05	9261	8	9624	1	5558	8	3975	08	1769	65	9045	74	3715	88	2097	44	5917	82	7418	21	9264	33	7571	36	9368	64	2208
**2001**	1450.369	51.29192	638.5157	24.37036	2216.808	85.90406	971.0295	35.59354	1877.435	65.14231	1415.275	53.43198	1574.525	58.69246	3282.817	131.525	1411.836	51.465	1447.394	57.04562	725.6619	28.33121	494.9539	19.81684	3220.88	130.1969	799.9322	30.35914	449.7892	18.13097
**2002**	1462.839	51.77633	644.5793	24.52006	2204.626	85.80626	993.0732	36.2145	1882.414	65.91945	1448.247	54.37083	1602.181	59.32272	3280.952	131.429	1433.604	52.56127	1477.669	58.10331	739.4927	28.84184	502.7689	20.09204	3226.799	130.2452	813.2848	30.74549	457.205	18.41278
**2003**	1474.847	52.27447	650.0412	24.63617	2189.402	85.59705	1016.436	36.90897	1886.652	66.88819	1483.858	55.32782	1628.064	59.97846	3274.211	130.9731	1454.309	53.7778	1508.184	59.25933	754.1981	29.38231	511.051	20.37839	3228.99	130.1346	827.1755	31.1583	465.9702	18.73779
**2004**	1485.63	52.72	654.7384	24.71	2171.78	85.28	1040.681	37.66	1891.249	67.87	1514.87	56.05	1651.612	60.69	3263.142	130.2	1473.481	54.94	1537.092	60.43	769.3426	29.92	518.7903	20.64	3230.442	130.0	841.7062	31.59	475.1596	19.07

		518		599		407		537		306		164		336		241		909		065		941		504		955		327		805
**2005**	1493.794	53.08321	658.5165	24.75684	2151.992	84.82965	1065.49	38.47273	1895.946	68.93217	1534.377	56.33468	1670.363	61.47593	3247.819	129.2179	1489.758	56.04078	1562.127	61.53694	784.4872	30.45994	524.9582	20.8594	3234.591	130.2559	857.084	32.04923	483.8717	19.404
**2006**	1499.546	53.38857	661.6848	24.77115	2111.192	83.55545	1099.594	39.63731	1900.194	70.09441	1545.56	56.33517	1684.898	62.46345	3209.445	127.331	1502.831	57.0719	1587.383	62.70997	803.5552	31.12589	531.0909	21.08797	3243.867	130.7374	880.5192	32.79323	493.0949	19.75147
**2007**	1504.376	53.70013	664.6709	24.77458	2044.403	81.30211	1145.82	41.25636	1904.537	71.31657	1555.224	56.30754	1697.822	63.71044	3142.678	124.4095	1514.507	58.10224	1616.593	64.06421	828.4351	32.00514	539.1589	21.39926	3257.099	131.4927	914.5609	33.91528	503.7792	20.14894
**2009**	1509.181	54.009	667.5266	24.771	1972.738	78.798	1194.328	42.997	1910.667	72.482	1564.354	56.268	1709.769	65.148	3068.459	121.35	1526.414	59.097	1646.41	65.476	855.6608	32.967	548.2125	21.751	3272.084	132.30	952.8086	35.178	514.8849	20.557

		61		51		71		54		05		99		06		07		25		16		38		64		44		48		58
**2008**	1515.303	54.35027	670.3346	24.76615	1916.919	76.78557	1238.237	44.60214	1918.612	73.68122	1573.31	56.23343	1722.648	66.72912	3008.708	119.0461	1539.137	60.12999	1675.017	66.84039	881.8721	33.88488	557.3359	22.10277	3286.238	132.9909	988.9848	36.36262	525.3657	20.93966
**2010**	1524.479	54.74197	673.1674	24.76403	1896.97	76.01326	1273.41	45.86542	1928.785	74.9046	1582.551	56.21229	1737.383	68.31543	2984.736	118.3404	1553.414	61.19433	1701.938	68.06499	903.8469	34.63229	565.7733	22.41078	3296.896	133.3994	1016.893	37.2375	534.1965	21.25838
**2011**	1537.272	55.1816	676.8687	24.80009	1905.791	76.28103	1300.703	46.80572	1941.403	76.07189	1595.148	56.32876	1756.873	69.87757	2993.702	118.9847	1569.627	62.22648	1728.886	69.16954	922.1928	35.2295	573.875	22.69277	3303.948	133.6233	1037.318	37.81607	542.2788	21.55524
**2012**	1551.929	55.6442	681.7459	24.8918	1921.302	76.8034	1325.389	47.6387	1954.46	77.0492	1610.953	56.6175	1781.579	71.5135	3013.521	120.089	1586.578	63.1660	1757.798	70.2505	939.7526	35.7923	582.4342	22.9926	3309.777	133.807	1055.567	38.2935	550.7577	21.8768

		3		9		6		6		7		1		5				3		2		3		3		1		2		9
**2013**	1567.271	56.11092	687.0516	25.01722	1941.159	77.5102	1348.385	48.41451	1967.17	77.79713	1627.248	56.97523	1809.327	73.16316	3038.987	121.4336	1603.86	63.99973	1787.574	71.28883	956.7033	36.33749	591.1676	23.29977	3315.211	133.9571	1072.356	38.71203	559.0061	22.19471
**2014**	1582.149	56.55969	692.0288	25.15169	1962.648	78.30819	1370.712	49.18929	1978.504	78.42894	1641.4	57.31811	1838.174	74.6775	3064.809	122.6605	1621.098	64.80364	1816.952	72.26853	973.2122	36.88108	599.6739	23.59886	3321.228	134.1312	1088.409	39.11368	566.3954	22.47968
**2015**	1595.281	56.97061	695.926	25.26912	1983.128	79.11231	1393.371	50.02136	1986.906	78.89874	1650.66	57.55321	1865.592	75.98967	3085.485	123.6117	1637.721	65.56313	1844.585	73.17661	989.3501	37.43424	607.7734	23.87935	3328.595	134.3406	1104.346	39.53592	572.2914	22.70374
**2016**	1608.988	57.4369	699.7921	25.36267	2021.215	80.55078	1420.476	51.09307	1991.609	79.04538	1625.392	57.0359	1896.741	77.30814	3121.873	125.2809	1656.58	66.33892	1865.581	73.89978	1007.187	38.07495	616.9063	24.15384	3338.009	134.8574	1122.163	40.0499	578.5349	22.87288

**2017**	1624.491	57.97335	703.8792	25.48116	2077.954	82.81732	1445.831	52.11745	1995.233	78.96849	1598.966	56.49324	1929.458	78.54829	3177.672	127.9844	1675.675	67.02573	1886.835	74.58557	1023.337	38.66595	625.6463	24.41318	3347.944	135.3676	1138.685	40.54176	585.4661	23.06391
**2018**	1647.139	58.72713	710.1907	25.75104	2158.559	86.05809	1467.24	52.96885	2007.89	79.05227	1607.289	56.77707	1964.366	79.80626	3256.04	131.5858	1697.479	67.71059	1920.761	75.68409	1039.35	39.24429	634.9722	24.72099	3365.409	136.0304	1154.287	41.03223	595.3635	23.44399
**2019**	1677.777	59.75754	720.1252	26.18631	2271.788	90.45831	1488.711	53.8415	2030.58	79.41043	1624.356	57.34561	2004.756	81.27976	3365.224	136.3264	1724.744	68.54218	1963.296	77.1514	1058.097	39.92715	646.7343	25.10932	3393.593	137.0998	1171.431	41.60413	609.8556	24.02451

S3 Table. Correlation analysis of EAPC with ASIR and ASPR.

location	case	EAPC	ASR	group
Afghanistan	2435.026	1.968146	31.3065	ASIR
Albania	104.5791	0.998188	5.456275	ASIR
Algeria	9645.619	1.621127	56.88011	ASIR
American Samoa	20.7223	1.091459	73.76597	ASIR
Andorra	22.63657	0.816717	107.6355	ASIR
Angola	852.7587	1.91928	13.54458	ASIR
Antigua and Barbuda	13.6937	0.880055	40.79667	ASIR
Argentina	7330.315	1.653708	41.61137	ASIR
Armenia	191.5236	1.747212	11.34488	ASIR
Australia	11816.49	0.538934	155.3643	ASIR
Austria	3676.27	0.045304	137.8405	ASIR
Azerbaijan	460.111	1.9142	11.4936	ASIR
Bahamas	82.79959	0.582522	53.47752	ASIR
Bahrain	194.6184	0.482589	81.65492	ASIR
Bangladesh	9200.999	2.135315	13.22025	ASIR
Barbados	66.38786	0.532024	52.46938	ASIR
Belarus	319.2574	1.25158	7.125022	ASIR
Belgium	4055.968	0.582611	113.2522	ASIR
Belize	52.52925	1.191162	40.9255	ASIR
Benin	451.4165	2.105505	15.66531	ASIR
Bermuda	14.629	0.241966	66.20727	ASIR
Bhutan	76.06782	2.455553	19.48732	ASIR
Bolivia (Plurinational State of)	3497.266	1.057218	83.52361	ASIR
Bosnia and Herzegovina	100.0272	1.842782	4.54026	ASIR
Botswana	230.9803	2.089673	24.31471	ASIR
Brazil	20680.82	-0.27828	22.30114	ASIR
Brunei Darussalam	137.0303	1.856257	96.50218	ASIR
Bulgaria	233.3236	0.892427	6.477654	ASIR
Burkina Faso	882.8639	1.43813	14.76357	ASIR
Burundi	499.8761	0.162866	14.8395	ASIR
Cabo Verde	38.39192	1.618334	17.07742	ASIR
Cambodia	2545.628	2.121348	39.13879	ASIR
Cameroon	1553.668	0.798605	23.69436	ASIR
Canada	5919.146	0.889616	54.05149	ASIR
Central African Republic	299.5926	0.011102	17.87512	ASIR
Chad	420.3291	1.024447	11.43738	ASIR
Chile	3253.888	2.177594	44.30639	ASIR
China	186317.1	2.293549	28.11011	ASIR
Colombia	11907.75	0.92163	58.9487	ASIR
Comoros	69.0814	0.347381	22.22214	ASIR
Congo	319.5609	1.005098	19.45653	ASIR
Cook Islands	8.777319	1.354653	76.41914	ASIR
Costa Rica	1334.327	0.964551	73.62226	ASIR
C 么 te d'Ivoire	1175.411	1.617344	15.78608	ASIR

Croatia	116.8539	1.265035	5.854123	ASIR
Cuba	2428.156	1.033761	45.09705	ASIR
Cyprus	288.9808	1.604269	82.24019	ASIR
Czechia	287.1807	0.906445	5.994658	ASIR
Democratic People's Republic of Korea	3408.145	0.279963	30.40048	ASIR
Democratic Republic of the Congo	3457.364	1.245156	14.67737	ASIR
Denmark	1930.594	0.872712	99.5337	ASIR
Djibouti	50.10112	2.065664	17.23183	ASIR
Dominica	17.9701	1.005438	40.83708	ASIR
Dominican Republic	1693.679	1.744015	35.24382	ASIR
Ecuador	7348.941	1.014622	109.812	ASIR
Egypt	23587.74	0.723253	69.84767	ASIR
El Salvador	2189.673	1.267396	59.2313	ASIR
Equatorial Guinea	40.78145	3.403448	15.70727	ASIR
Eritrea	241.4287	1.392912	12.79707	ASIR
Estonia	49.5106	1.601282	7.640426	ASIR
Eswatini	189.1021	0.522524	32.40305	ASIR
Ethiopia	4117.484	1.578861	13.3253	ASIR
Fiji	265.3988	1.457348	56.52619	ASIR
Finland	1774.846	0.849469	99.44652	ASIR
France	21095.99	0.825611	90.94926	ASIR
Gabon	136.0246	1.345187	21.93664	ASIR
Gambia	99.50717	1.131373	15.87468	ASIR
Georgia	361.5767	2.485767	14.52498	ASIR
Germany	22539.04	0.674673	93.21811	ASIR
Ghana	1534.693	1.101064	16.5016	ASIR
Greece	4714.145	0.721731	108.2311	ASIR
Greenland	9.58223	1.15474	44.94195	ASIR
Grenada	18.06759	1.130738	34.20499	ASIR
Guam	55.57634	1.319151	82.11776	ASIR
Guatemala	2645.345	1.129261	49.81309	ASIR
Guinea	504.9167	1.233089	14.33808	ASIR
Guinea-Bissau	94.02897	1.220107	14.40264	ASIR
Guyana	185.0257	1.27849	35.64591	ASIR
Haiti	1059.466	0.909552	25.93818	ASIR
Honduras	1555.747	1.571683	47.30202	ASIR
Hungary	303.2313	0.802759	6.600228	ASIR
Iceland	131.5547	0.940734	111.6758	ASIR
India	122844.9	2.241091	25.06246	ASIR
Indonesia	60831.96	2.691267	51.32823	ASIR
Iran (Islamic Republic of)	22820.99	1.719606	59.93677	ASIR
Iraq	8034.481	0.639856	68.15225	ASIR
Ireland	2023.548	0.830115	103.1871	ASIR
Israel	2453.865	0.900236	90.40184	ASIR
Italy	73673.08	-0.63845	344.512	ASIR
Jamaica	596.2798	1.082033	39.19852	ASIR

**Japan**	137816.5	0.285296	242.4785	ASIR
**Jordan**	1647.868	1.056478	62.70024	ASIR
**Kazakhstan**	1117.735	1.378038	12.92257	ASIR
**Kenya**	3212.911	0.476821	20.41312	ASIR
**Kiribati**	20.04579	1.294627	47.39184	ASIR
**Kuwait**	775.4555	0.966924	89.2195	ASIR
**Kyrgyzstan**	302.6198	0.536607	11.78836	ASIR
**Lao People's Democratic Republic**	1209.382	2.480256	44.55186	ASIR
**Latvia**	81.34564	1.16878	7.623948	ASIR
**Lebanon**	1219.598	1.032207	67.69545	ASIR
**Lesotho**	262.3804	1.490222	21.4444	ASIR
**Liberia**	207.5528	1.532607	16.40271	ASIR
**Libya**	2337.976	0.542574	78.66229	ASIR
**Lithuania**	112.5684	1.410373	6.978692	ASIR
**Luxembourg**	128.6306	0.984079	105.3447	ASIR
**Madagascar**	1238.706	0.552181	16.13665	ASIR
**Malawi**	1373.873	0.606568	22.18817	ASIR
**Malaysia**	9441.729	2.227138	88.43735	ASIR
**Maldives**	69.12427	3.997967	47.95914	ASIR
**Mali**	618.8426	1.391159	12.25665	ASIR
**Malta**	157.5501	1.155006	97.77915	ASIR
**Marshall Islands**	12.53386	1.701571	39.91656	ASIR
**Mauritania**	264.214	0.829441	20.5865	ASIR
**Mauritius**	542.8442	1.886513	86.78891	ASIR
**Mexico**	79250.35	-0.61065	132.8926	ASIR
**Micronesia (Federated States of)**	38.39913	1.153776	53.38368	ASIR
**Monaco**	8.035105	0.631773	116.3609	ASIR
**Mongolia**	157.4098	1.386019	11.34391	ASIR
**Montenegro**	19.16581	1.109483	6.336946	ASIR
**Morocco**	9391.737	1.101158	57.43619	ASIR
**Mozambique**	1361.614	1.491596	15.76309	ASIR
**Myanmar**	10482.49	3.216139	41.08316	ASIR
**Namibia**	224.2145	1.058872	23.26426	ASIR
**Nauru**	3.808201	1.160865	62.57703	ASIR
**Nepal**	1596.049	2.119162	13.51489	ASIR
**Netherlands**	5175.27	0.822978	91.62911	ASIR
**New Zealand**	3684.62	-0.28115	227.4746	ASIR
**Nicaragua**	1529.116	1.260975	54.80331	ASIR
**Niger**	643.4482	0.991893	12.88082	ASIR
**Nigeria**	10698.85	0.771089	18.42691	ASIR
**Niue**	0.854126	1.541853	69.04331	ASIR
**North Macedonia**	52.30045	1.31751	5.258537	ASIR
**Northern Mariana Islands**	18.8187	0.795312	78.37378	ASIR
**Norway**	2087.04	0.318172	128.3004	ASIR
**Oman**	573.8967	2.099009	54.07382	ASIR
**Pakistan**	16688.76	0.759249	24.51794	ASIR
**Palau**	6.432727	1.249405	72.69465	ASIR

Palestine	770.8185	0.859622	56.96543	ASIR
Panama	726.9049	1.570866	49.55839	ASIR
Papua New Guinea	952.9017	1.180346	37.99866	ASIR
Paraguay	332.407	1.901422	13.52012	ASIR
Peru	12721.23	1.117997	89.44033	ASIR
Philippines	23263.76	2.100429	56.85935	ASIR
Poland	1662.635	0.091636	9.548875	ASIR
Portugal	4213.55	0.940019	87.36946	ASIR
Puerto Rico	1256.709	0.928104	64.72594	ASIR
Qatar	148.3999	0.679727	88.5946	ASIR
Republic of Korea	20316.45	1.269017	79.41283	ASIR
Republic of Moldova	127.3844	1.559204	6.038463	ASIR
Romania	618.249	1.361167	5.488729	ASIR
Russian Federation	5063.708	1.051293	7.747736	ASIR
Rwanda	918.1276	0.965154	19.83225	ASIR
Saint Kitts and Nevis	11.90254	1.017087	46.94882	ASIR
Saint Lucia	35.0821	0.808298	38.43351	ASIR
Saint Vincent and the Grenadines	25.9072	1.593028	34.57213	ASIR
Samoa	73.33181	0.980217	64.67585	ASIR
San Marino	11.38293	0.690046	110.7842	ASIR
Sao Tome and Principe	14.06082	1.170758	17.42186	ASIR
Saudi Arabia	6895.787	1.427265	70.79085	ASIR
Senegal	931.312	0.557268	18.56444	ASIR
Serbia	229.657	1.255743	5.488729	ASIR
Seychelles	40.6919	1.17027	92.91235	ASIR
Sierra Leone	308.9114	1.699572	14.12902	ASIR
Singapore	1288.912	1.93654	90.34444	ASIR
Slovakia	144.4387	1.171244	5.64892	ASIR
Slovenia	51.66803	1.288484	6.085624	ASIR
Solomon Islands	91.19097	1.4584	39.50415	ASIR
Somalia	717.7273	0.695397	16.06463	ASIR
South Africa	7786.274	1.075042	32.28578	ASIR
South Sudan	663.2417	0.195392	17.80722	ASIR
Spain	18458.18	0.69753	101.2299	ASIR
Sri Lanka	6914.427	2.23824	67.88995	ASIR
Sudan	4696.419	2.18256	36.06112	ASIR
Suriname	91.6109	1.126909	40.62448	ASIR
Sweden	2293.688	0.441332	76.04359	ASIR
Switzerland	2279.215	0.5074	102.1114	ASIR
Syrian Arab Republic	5184.219	1.149743	57.89655	ASIR
Taiwan (Province of China)	6111.952	2.051341	56.73654	ASIR
Tajikistan	305.979	0.985272	9.391518	ASIR
Thailand	21871.8	2.625405	64.00337	ASIR
Timor-Leste	176.3543	2.224286	42.09817	ASIR
Togo	350.7605	1.197995	14.83848	ASIR
Tokelau	0.535486	1.719	56.67997	ASIR
Tonga	50.16896	0.84596	74.37121	ASIR

**Trinidad and Tobago**	276.3832	1.531939	40.53838	ASIR
**Tunisia**	2894.682	1.458188	55.22874	ASIR
**Turkey**	20070.93	1.486397	53.39599	ASIR
**Turkmenistan**	274.4568	1.391025	12.17899	ASIR
**Tuvalu**	2.470638	1.479666	52.12797	ASIR
**Uganda**	2094.402	0.830193	18.25117	ASIR
**Ukraine**	1630.251	0.848414	7.290746	ASIR
**United Arab Emirates**	522.3606	1.048016	71.1914	ASIR
**United Kingdom**	23755.57	0.59725	113.4631	ASIR
**United Republic of Tanzania**	3777.343	0.695002	21.37125	ASIR
**United States of America**	122177.1	-0.93914	119.0695	ASIR
**United States Virgin Islands**	36.29873	0.845645	62.19508	ASIR
**Uruguay**	647.1384	1.950422	42.62368	ASIR
**Uzbekistan**	1570.182	1.261379	12.40766	ASIR
**Vanuatu**	44.15548	1.355216	46.87001	ASIR
**Venezuela (Bolivarian Republic of)**	8600.503	0.786813	71.50304	ASIR
**Viet Nam**	16681.89	3.195164	38.75228	ASIR
**Yemen**	3226.36	1.177957	37.17842	ASIR
**Zambia**	1429.839	0.581275	24.83851	ASIR
**Zimbabwe**	1985.474	-0.07235	26.67053	ASIR
**Afghanistan**	41088.95	2.004196	808.4342	ASPR
**Albania**	2300.138	1.002386	137.7578	ASPR
**Algeria**	174971.8	1.642063	1471.314	ASPR
**American Samoa**	470.7721	1.089534	1941.241	ASPR
**Andorra**	829.8078	0.791634	2771.955	ASPR
**Angola**	15552.46	2.013337	339.6166	ASPR
**Antigua and Barbuda**	349.2118	0.889686	1069.301	ASPR
**Argentina**	170766.7	1.650684	1055.527	ASPR
**Armenia**	5041.281	1.756403	284.8723	ASPR
**Australia**	326245.9	0.527629	3646.411	ASPR
**Austria**	142685.5	0.045184	3602.484	ASPR
**Azerbaijan**	11052.94	1.985973	289.8149	ASPR
**Bahamas**	2054.131	0.58978	1406.241	ASPR
**Bahrain**	5014.8	0.481694	2136.184	ASPR
**Bangladesh**	161923.1	2.217835	327.0685	ASPR
**Barbados**	1895.522	0.536457	1378.437	ASPR
**Belarus**	9211.853	1.301971	175.9056	ASPR
**Belgium**	143927.4	0.599222	2935.941	ASPR
**Belize**	907.0251	1.211538	1056.951	ASPR
**Benin**	8545.265	2.174734	391.1174	ASPR
**Bermuda**	594.915	0.241874	1743.547	ASPR
**Bhutan**	1374.765	2.535619	489.73	ASPR
**Bolivia (Plurinational State of)**	63843.75	1.162371	2062.158	ASPR
**Bosnia and Herzegovina**	2718.327	1.881981	113.6536	ASPR
**Botswana**	3983.42	2.133765	615.9847	ASPR
**Brazil**	414242.2	-0.10249	531.1987	ASPR
**Brunei Darussalam**	3154.699	1.816137	2324.805	ASPR

**Bulgaria**	6889.274	0.903861	164.4014	ASPR
**Burkina Faso**	15364.22	1.550552	363.4555	ASPR
**Burundi**	9212.427	0.126328	367.5595	ASPR
**Cabo Verde**	685.3349	1.674663	432.797	ASPR
**Cambodia**	50499.23	2.104909	1008.425	ASPR
**Cameroon**	28984.29	0.822689	607.5077	ASPR
**Canada**	201395	0.891951	1351.818	ASPR
**Central African Republic**	5893.195	-0.00997	458.711	ASPR
**Chad**	7402.109	1.107894	282.0723	ASPR
**Chile**	81708.99	2.173142	1127.326	ASPR
**China**	4797942	2.387361	750.0965	ASPR
**Colombia**	263405.6	0.988847	1502.829	ASPR
**Comoros**	1203.407	0.397293	572.6873	ASPR
**Congo**	5559.456	1.013113	494.6139	ASPR
**Cook Islands**	185.7567	1.342364	2011.693	ASPR
**Costa Rica**	30381.56	0.96353	1928.993	ASPR
**C 么 te d'Ivoire**	21498.71	1.702256	394.9175	ASPR
**Croatia**	3628.881	1.282789	148.4855	ASPR
**Cuba**	71869.05	1.044758	1182.201	ASPR
**Cyprus**	8457.558	1.55114	2127.868	ASPR
**Czechia**	7924.335	0.918991	152.9806	ASPR
**Democratic People's Republic of Korea**	88649.37	0.275332	788.8547	ASPR
**Democratic Republic of the Congo**	62493.53	1.317762	367.9065	ASPR
**Denmark**	63247.83	0.948793	2421.297	ASPR
**Djibouti**	917.589	2.144418	440.4584	ASPR
**Dominica**	368.2612	1.022876	1069.475	ASPR
**Dominican Republic**	34588.85	1.778154	909.221	ASPR
**Ecuador**	139942	1.169243	2734.522	ASPR
**Egypt**	484497.9	0.745368	1821.166	ASPR
**El Salvador**	40427.2	1.332082	1523.473	ASPR
**Equatorial Guinea**	777.8218	3.532753	396.9304	ASPR
**Eritrea**	4309.667	1.497386	318.1144	ASPR
**Estonia**	1484.645	1.681202	189.6808	ASPR
**Eswatini**	3238.723	0.511133	825.383	ASPR
**Ethiopia**	72350.8	1.733742	322.9825	ASPR
**Fiji**	5854.874	1.462252	1482.585	ASPR
**Finland**	62901.41	0.875139	2468.67	ASPR
**France**	685273.8	0.839571	2362.924	ASPR
**Gabon**	2479.439	1.345211	565.1768	ASPR
**Gambia**	1799.683	1.198442	399.5092	ASPR
**Georgia**	10382.18	2.518936	370.2287	ASPR
**Germany**	911642.7	0.708217	2315.299	ASPR
**Ghana**	29068.79	1.163771	412.022	ASPR
**Greece**	141334.3	0.690351	2783.427	ASPR
**Greenland**	338.4647	1.159457	1120.118	ASPR
**Grenada**	351.564	1.146439	890.6985	ASPR

Guam	1515.343	1.309833	2160.705	ASPR
Guatemala	47089.62	1.169405	1288.999	ASPR
Guinea	10114.31	1.284267	359.2251	ASPR
Guinea-Bissau	1695.92	1.29871	359.8592	ASPR
Guyana	3835.543	1.297165	931.8959	ASPR
Haiti	20789.33	0.922937	669.3766	ASPR
Honduras	26549.63	1.559452	1232.662	ASPR
Hungary	8601.348	0.813776	167.5879	ASPR
Iceland	3755.564	0.90476	2883.229	ASPR
India	2565477	2.625673	631.8998	ASPR
Indonesia	1280947	2.688782	1331.196	ASPR
Iran (Islamic Republic of)	415825.9	1.695208	1568.521	ASPR
Iraq	138294.3	0.649959	1775.824	ASPR
Ireland	47132.7	0.809166	2658.067	ASPR
Israel	56640.42	0.872268	2305.778	ASPR
Italy	2346824	-0.41435	8223.579	ASPR
Jamaica	12393.31	1.097895	1029.355	ASPR
Japan	3821778	0.202005	5905.757	ASPR
Jordan	27455.31	1.069571	1606.272	ASPR
Kazakhstan	27452.52	1.428979	326.5785	ASPR
Kenya	51323.8	0.659176	505.3459	ASPR
Kiribati	467.3254	1.302427	1240.304	ASPR
Kuwait	20058.08	0.963515	2335.314	ASPR
Kyrgyzstan	6332.114	0.502061	297.8431	ASPR
Lao People's Democratic Republic	22330.9	2.498599	1147.948	ASPR
Latvia	2506.803	1.223068	188.6869	ASPR
Lebanon	27519.35	1.04065	1778.242	ASPR
Lesotho	4650.863	1.523626	546.7675	ASPR
Liberia	3598.344	1.633838	413.8049	ASPR
Libya	36793.28	0.565122	2036.931	ASPR
Lithuania	3231.448	1.48065	171.9697	ASPR
Luxembourg	5314.553	0.954835	2711.535	ASPR
Madagascar	21626.95	0.593492	404.1921	ASPR
Malawi	25071.25	0.594797	570.2691	ASPR
Malaysia	206933.6	2.22043	2291.722	ASPR
Maldives	1202.671	4.00118	1253.153	ASPR
Mali	11501.19	1.514637	301.1521	ASPR
Malta	4802.373	1.117398	2520.961	ASPR
Marshall Islands	207.524	1.723446	1041.324	ASPR
Mauritania	4880.877	0.918386	518.8455	ASPR
Mauritius	13649.03	1.875216	2247.644	ASPR
Mexico	1503173	-0.59549	3404.15	ASPR
Micronesia (Federated States of)	655.6515	1.159288	1399.236	ASPR
Monaco	418.0841	0.609349	2993.816	ASPR
Mongolia	2921.289	1.434564	285.3748	ASPR
Montenegro	510.5151	1.126156	161.382	ASPR
Morocco	189955.9	1.182671	1485.742	ASPR

Mozambique	24392.36	1.536826	398.8866	ASPR
Myanmar	216304.5	3.240632	1055.449	ASPR
Namibia	3981.346	1.031876	588.701	ASPR
Nauru	80.65201	1.161324	1644.487	ASPR
Nepal	30682.39	2.174409	335.3747	ASPR
Netherlands	187340.1	0.802693	2364.322	ASPR
New Zealand	96861.22	-0.17394	5357.519	ASPR
Nicaragua	25962.22	1.281428	1415.633	ASPR
Niger	11016.68	1.021964	320.425	ASPR
Nigeria	180357.6	0.927434	455.3914	ASPR
Niue	18.28979	1.535057	1816.21	ASPR
North Macedonia	1374.838	1.353276	132.4317	ASPR
Northern Mariana Islands	568.5627	0.793361	2061.48	ASPR
Norway	69416.46	0.294857	3293.699	ASPR
Oman	9817.247	2.105717	1410.849	ASPR
Pakistan	288444.1	0.517634	611.7515	ASPR
Palau	157.7228	1.244837	1911.354	ASPR
Palestine	13263.67	0.870543	1482.757	ASPR
Panama	16055.83	1.568552	1301.323	ASPR
Papua New Guinea	19129.64	1.206293	984.6521	ASPR
Paraguay	6532.296	1.955193	348.5963	ASPR
Peru	234444.5	1.379303	2131.357	ASPR
Philippines	458271.6	2.3568	1473.264	ASPR
Poland	46395.23	0.1671	242.1234	ASPR
Portugal	114851.1	0.907269	2255.355	ASPR
Puerto Rico	32801.93	0.930977	1704.011	ASPR
Qatar	3713.264	0.677955	2316.931	ASPR
Republic of Korea	488973.7	1.263573	1924.881	ASPR
Republic of Moldova	3388.187	1.644204	146.9101	ASPR
Romania	15839.76	1.405552	138.3123	ASPR
Russian Federation	145531.3	1.104411	187.1103	ASPR
Rwanda	15721.56	0.926376	486.5921	ASPR
Saint Kitts and Nevis	249.1308	1.025313	1230.324	ASPR
Saint Lucia	692.7108	0.818816	1004.998	ASPR
Saint Vincent and the Grenadines	481.33	1.61816	901.6625	ASPR
Samoa	1265.993	0.976725	1697.829	ASPR
San Marino	337.6156	0.665694	2850.32	ASPR
Sao Tome and Principe	221.4453	1.210395	431.8186	ASPR
Saudi Arabia	123981.1	1.434636	1847.958	ASPR
Senegal	16103.52	0.588027	468.9439	ASPR
Serbia	6379.027	1.273439	138.3077	ASPR
Seychelles	875.8863	1.162537	2401.771	ASPR
Sierra Leone	6106.877	1.77723	353.7496	ASPR
Singapore	41302.5	1.892462	2181.322	ASPR
Slovakia	3827.094	1.185796	142.8005	ASPR
Slovenia	1530.576	1.357238	151.8963	ASPR
Solomon Islands	1549.418	1.475163	1029.314	ASPR

Somalia	12423.86	0.720987	405.0643	ASPR
South Africa	162944.4	1.111306	832.7065	ASPR
South Sudan	11514.26	0.205528	451.3358	ASPR
Spain	477831.9	0.830596	2471.312	ASPR
Sri Lanka	161955.5	2.234931	1751.477	ASPR
Sudan	88711.77	2.232571	937.0061	ASPR
Suriname	2071.582	1.133739	1062.731	ASPR
Sweden	81951.41	0.396415	1992.852	ASPR
Switzerland	92700.22	0.492402	2625.73	ASPR
Syrian Arab Republic	84784.15	1.166279	1500.234	ASPR
Taiwan (Province of China)	164859.7	2.042067	1484.342	ASPR
Tajikistan	5663.702	1.01305	233.9872	ASPR
Thailand	529398.1	2.632651	1645.866	ASPR
Timor-Leste	4048.321	2.227969	1081.514	ASPR
Togo	6196.125	1.317298	365.3642	ASPR
Tokelau	10.57048	1.72498	1487.92	ASPR
Tonga	888.904	0.839334	1957.944	ASPR
Trinidad and Tobago	6552.045	1.565966	1054.771	ASPR
Tunisia	59897.63	1.457495	1439.106	ASPR
Turkey	412515	1.526226	1380.555	ASPR
Turkmenistan	5481.83	1.439765	307.1408	ASPR
Tuvalu	66.87023	1.488108	1367.693	ASPR
Uganda	35243.89	0.852681	461.2129	ASPR
Ukraine	46492.59	0.896231	174.7675	ASPR
United Arab Emirates	12970.9	1.050423	1859.141	ASPR
United Kingdom	838385.8	0.578475	2929.818	ASPR
United Republic of Tanzania	65611.3	0.729563	549.5575	ASPR
United States of America	4005172	-0.86684	2962.638	ASPR
United States Virgin Islands	943.7534	0.846702	1639.334	ASPR
Uruguay	16403.6	1.944867	1082.596	ASPR
Uzbekistan	30886.72	1.294739	312.6139	ASPR
Vanuatu	868.1527	1.363822	1226.895	ASPR
Venezuela (Bolivarian Republic of)	183380.3	0.784606	1868.689	ASPR
Viet Nam	337635.6	3.212325	983.084	ASPR
Yemen	54198.68	1.193936	965.323	ASPR
Zambia	23321.66	0.557099	636.5316	ASPR
Zimbabwe	32678.84	-0.12733	677.9055	ASPR

S4 Table. Correlation analysis of EAPC with HDI.

location	case	EAPC	HDI	group
Afghanistan	11947.85	1.968146	0.498	ASIR
Albania	73.97199	0.998188	0.785	ASIR
Algeria	15931.7	1.621127	0.754	ASIR
Andorra	32.53313	0.816717	0.858	ASIR
Angola	4956.007	1.91928	0.581	ASIR
Antigua and Barbuda	20.77962	0.880055	0.78	ASIR
Argentina	13848.29	1.653708	0.825	ASIR
Armenia	185.3281	1.747212	0.755	ASIR
Australia	16077.26	0.538934	0.939	ASIR
Austria	3644.162	0.045304	0.908	ASIR
Azerbaijan	761.4755	1.9142	0.757	ASIR
Bahamas	117.3414	0.582522	0.807	ASIR
Bahrain	448.3335	0.482589	0.846	ASIR
Bangladesh	20554.91	2.135315	0.608	ASIR
Barbados	67.92771	0.532024	0.8	ASIR
Belarus	291.0444	1.25158	0.808	ASIR
Belgium	5019.165	0.582611	0.916	ASIR
Belize	157.8429	1.191162	0.708	ASIR
Benin	2457.807	2.105505	0.515	ASIR
Bhutan	145.8422	2.455553	0.612	ASIR
Botswana	501.9724	2.089673	0.717	ASIR
Brazil	23141.07	-0.27828	0.759	ASIR
Bulgaria	162.1064	0.892427	0.813	ASIR
Burundi	1270.643	0.162866	0.417	ASIR
Cambodia	6081.035	2.121348	0.582	ASIR
Cameroon	6288.943	0.798605	0.556	ASIR
Canada	8307.267	0.889616	0.926	ASIR
Chad	1907.645	1.024447	0.404	ASIR
Chile	5777.359	2.177594	0.843	ASIR
China	223654.3	2.293549	0.752	ASIR
Colombia	17282.71	0.92163	0.747	ASIR
Comoros	112.7229	0.347381	0.503	ASIR
Congo	873.1146	1.005098	0.457	ASIR
Croatia	102.8561	1.265035	0.831	ASIR
Cuba	2174.282	1.033761	0.777	ASIR
Cyprus	474.0269	1.604269	0.869	ASIR
Denmark	2484.256	0.872712	0.929	ASIR
Djibouti	174.8657	2.065664	0.476	ASIR
Dominica	17.97435	1.005438	0.715	ASIR
Ecuador	13624.73	1.014622	0.752	ASIR
Egypt	49168.8	0.723253	0.696	ASIR
Eritrea	868.0925	1.392912	0.44	ASIR
Estonia	45.34022	1.601282	0.871	ASIR
Ethiopia	14568.83	1.578861	0.463	ASIR

**Fiji**	416.1907	1.457348	0.741	ASIR
**Finland**	2170.175	0.849469	0.92	ASIR
**France**	27341.11	0.825611	0.901	ASIR
**Gabon**	368.8418	1.345187	0.702	ASIR
**Georgia**	318.8248	2.485767	0.78	ASIR
**Germany**	25684.2	0.674673	0.936	ASIR
**Ghana**	4847.317	1.101064	0.592	ASIR
**Greece**	4162.101	0.721731	0.87	ASIR
**Grenada**	23.10427	1.130738	0.772	ASIR
**Guatemala**	7742.66	1.129261	0.65	ASIR
**Guinea**	1825.922	1.233089	0.459	ASIR
**Guinea-Bissau**	277.017	1.220107	0.455	ASIR
**Guyana**	211.1171	1.27849	0.654	ASIR
**Haiti**	2462.613	0.909552	0.498	ASIR
**Honduras**	4544.02	1.571683	0.617	ASIR
**Hungary**	240.7491	0.802759	0.838	ASIR
**Iceland**	180.4696	0.940734	0.935	ASIR
**India**	332699.8	2.241091	0.64	ASIR
**Indonesia**	135161.6	2.691267	0.694	ASIR
**Iraq**	19121.08	0.639856	0.685	ASIR
**Ireland**	2504.431	0.830115	0.938	ASIR
**Israel**	5092.144	0.900236	0.903	ASIR
**Italy**	50089.92	-0.63845	0.88	ASIR
**Jamaica**	732.1097	1.082033	0.732	ASIR
**Japan**	89573.34	0.285296	0.909	ASIR
**Jordan**	5486.296	1.056478	0.735	ASIR
**Kazakhstan**	1510.788	1.378038	0.8	ASIR
**Kenya**	8264.853	0.476821	0.59	ASIR
**Kiribati**	50.61894	1.294627	0.612	ASIR
**Kuwait**	1566.338	0.966924	0.803	ASIR
**Kyrgyzstan**	480.5047	0.536607	0.672	ASIR
**Latvia**	58.86657	1.16878	0.847	ASIR
**Lebanon**	1859.549	1.032207	0.757	ASIR
**Lesotho**	424.4824	1.490222	0.52	ASIR
**Liberia**	785.7372	1.532607	0.435	ASIR
**Libya**	2805.018	0.542574	0.706	ASIR
**Lithuania**	83.64359	1.410373	0.858	ASIR
**Luxembourg**	262.7419	0.984079	0.904	ASIR
**Madagascar**	3284.09	0.552181	0.519	ASIR
**Malawi**	3577.661	0.606568	0.477	ASIR
**Malaysia**	23897.59	2.227138	0.802	ASIR
**Maldives**	210.7923	3.997967	0.717	ASIR
**Mali**	2894.654	1.391159	0.427	ASIR
**Malta**	159.434	1.155006	0.878	ASIR
**Mauritania**	813.3603	0.829441	0.52	ASIR
**Mauritius**	719.2319	1.886513	0.79	ASIR
**Mexico**	84013.67	-0.61065	0.774	ASIR

Mongolia	241.1483	1.386019	0.741	ASIR
Montenegro	18.81301	1.109483	0.814	ASIR
Morocco	13917.3	1.101158	0.667	ASIR
Mozambique	5008.61	1.491596	0.437	ASIR
Namibia	463.9921	1.058872	0.647	ASIR
Nepal	4616.634	2.119162	0.574	ASIR
Netherlands	6599.599	0.822978	0.931	ASIR
Nicaragua	2943.429	1.260975	0.658	ASIR
Niger	2729.874	0.991893	0.354	ASIR
Nigeria	37538.24	0.771089	0.532	ASIR
North Macedonia	53.24924	1.31751	0.757	ASIR
Norway	2463.958	0.318172	0.953	ASIR
Oman	1435.752	2.099009	0.821	ASIR
Pakistan	40418.63	0.759249	0.562	ASIR
Palau	7.308747	1.249405	0.798	ASIR
Palestine	2280.842	0.859622	0.686	ASIR
Panama	1692.538	1.570866	0.789	ASIR
Papua New Guinea	3125.885	1.180346	0.544	ASIR
Paraguay	823.3899	1.901422	0.702	ASIR
Peru	19700.24	1.117997	0.75	ASIR
Philippines	61096.97	2.100429	0.699	ASIR
Poland	1113.421	0.091636	0.865	ASIR
Portugal	3840.422	0.940019	0.847	ASIR
Qatar	634.1237	0.679727	0.856	ASIR
Romania	491.1562	1.361167	0.811	ASIR
Rwanda	2145.669	0.965154	0.524	ASIR
Saint Kitts and Nevis	16.45341	1.017087	0.778	ASIR
Saint Lucia	38.30807	0.808298	0.747	ASIR
Saint Vincent and the Grenadines	27.56457	1.593028	0.723	ASIR
Samoa	124.8181	0.980217	0.713	ASIR
Sao Tome and Principe	35.88551	1.170758	0.589	ASIR
Saudi Arabia	13508.9	1.427265	0.853	ASIR
Senegal	2293.595	0.557268	0.505	ASIR
Serbia	222.5156	1.255743	0.787	ASIR
Seychelles	53.69605	1.17027	0.797	ASIR
Sierra Leone	1369.663	1.699572	0.419	ASIR
Singapore	2136.357	1.93654	0.932	ASIR
Slovakia	130.6582	1.171244	0.855	ASIR
Slovenia	49.31415	1.288484	0.896	ASIR
Solomon Islands	256.2148	1.4584	0.546	ASIR
South Africa	11757.2	1.075042	0.699	ASIR
South Sudan	1280.855	0.195392	0.388	ASIR
Spain	17370.71	0.69753	0.891	ASIR
Sri Lanka	12379.48	2.23824	0.77	ASIR
Sudan	17975.97	2.18256	0.502	ASIR
Suriname	154.8798	1.126909	0.72	ASIR

Sweden	3111.885	0.441332	0.933	ASIR
Switzerland	2851.57	0.5074	0.944	ASIR
Tajikistan	655.0942	0.985272	0.65	ASIR
Thailand	31480.67	2.625405	0.755	ASIR
Timor-Leste	661.0023	2.224286	0.625	ASIR
Togo	1112.613	1.197995	0.503	ASIR
Tonga	65.31575	0.84596	0.726	ASIR
Trinidad and Tobago	324.8159	1.531939	0.784	ASIR
Tunisia	3799.437	1.458188	0.735	ASIR
Turkey	26634.28	1.486397	0.791	ASIR
Turkmenistan	437.3136	1.391025	0.706	ASIR
Uganda	6745.681	0.830193	0.516	ASIR
Ukraine	1308.211	0.848414	0.751	ASIR
United Arab Emirates	2008.557	1.048016	0.863	ASIR
United Kingdom	31646	0.59725	0.922	ASIR
Uruguay	1048.732	1.950422	0.804	ASIR
Uzbekistan	3142.441	1.261379	0.71	ASIR
Vanuatu	125.7103	1.355216	0.603	ASIR
Yemen	9792.336	1.177957	0.452	ASIR
Zambia	3914.413	0.581275	0.588	ASIR
Zimbabwe	2918.123	-0.07235	0.535	ASIR
Afghanistan	216686.1	2.004196	0.498	ASPR
Albania	2417.334	1.002386	0.785	ASPR
Algeria	499279.4	1.642063	0.754	ASPR
Andorra	1417.897	0.791634	0.858	ASPR
Angola	91163.57	2.013337	0.581	ASPR
Antigua and Barbuda	724.3142	0.889686	0.78	ASPR
Argentina	404209.1	1.650684	0.825	ASPR
Armenia	7143.016	1.756403	0.755	ASPR
Australia	527760.8	0.527629	0.939	ASPR
Austria	155692.5	0.045184	0.908	ASPR
Azerbaijan	27426.81	1.985973	0.757	ASPR
Bahamas	3585.608	0.58978	0.807	ASPR
Bahrain	16239.06	0.481694	0.846	ASPR
Bangladesh	517126	2.217835	0.608	ASPR
Barbados	2375.787	0.536457	0.8	ASPR
Belarus	11362.27	1.301971	0.808	ASPR
Belgium	181470.1	0.599222	0.916	ASPR
Belize	3691.506	1.211538	0.708	ASPR
Benin	45847.47	2.174734	0.515	ASPR
Bhutan	3896.287	2.535619	0.612	ASPR
Botswana	13549.68	2.133765	0.717	ASPR
Brazil	703964	-0.10249	0.759	ASPR
Bulgaria	6359.438	0.903861	0.813	ASPR
Burundi	22225.02	0.126328	0.417	ASPR
Cambodia	162280	2.104909	0.582	ASPR
Cameroon	122535.2	0.822689	0.556	ASPR

**Canada**	290955.5	0.891951	0.926	ASPR
**Chad**	31362.46	1.107894	0.404	ASPR
**Chile**	193502.6	2.173142	0.843	ASPR
**China**	10550679	2.387361	0.752	ASPR
**Colombia**	529828.3	0.988847	0.747	ASPR
**Comoros**	2565.288	0.397293	0.503	ASPR
**Congo**	19082.56	1.013113	0.457	ASPR
**Croatia**	3854.092	1.282789	0.831	ASPR
**Cuba**	84003.85	1.044758	0.777	ASPR
**Cyprus**	22770.53	1.55114	0.869	ASPR
**Denmark**	81693.53	0.948793	0.929	ASPR
**Djibouti**	4865.43	2.144418	0.476	ASPR
**Dominica**	505.4117	1.022876	0.715	ASPR
**Ecuador**	342433.2	1.169243	0.752	ASPR
**Egypt**	1192757	0.745368	0.696	ASPR
**Eritrea**	17034.66	1.497386	0.44	ASPR
**Estonia**	1668.85	1.681202	0.871	ASPR
**Ethiopia**	265653	1.733742	0.463	ASPR
**Fiji**	10978.76	1.462252	0.741	ASPR
**Finland**	74322.66	0.875139	0.92	ASPR
**France**	882322.9	0.839571	0.901	ASPR
**Gabon**	8592.812	1.345211	0.702	ASPR
**Georgia**	11728.96	2.518936	0.78	ASPR
**Germany**	1028528	0.708217	0.936	ASPR
**Ghana**	110954.9	1.163771	0.592	ASPR
**Greece**	162493	0.690351	0.87	ASPR
**Grenada**	699.6188	1.146439	0.772	ASPR
**Guatemala**	190094.5	1.169405	0.65	ASPR
**Guinea**	34758.62	1.284267	0.459	ASPR
**Guinea-Bissau**	5584.456	1.29871	0.455	ASPR
**Guyana**	5826.992	1.297165	0.654	ASPR
**Haiti**	60009.57	0.922937	0.498	ASPR
**Honduras**	106563.3	1.559452	0.617	ASPR
**Hungary**	9430.964	0.813776	0.838	ASPR
**Iceland**	5884.468	0.90476	0.935	ASPR
**India**	9157101	2.625673	0.64	ASPR
**Indonesia**	3840203	2.688782	0.694	ASPR
**Iraq**	455161.7	0.649959	0.685	ASPR
**Ireland**	80770.41	0.809166	0.938	ASPR
**Israel**	133195	0.872268	0.903	ASPR
**Italy**	1981391	-0.41435	0.88	ASPR
**Jamaica**	22423.96	1.097895	0.732	ASPR
**Japan**	3271230	0.202005	0.909	ASPR
**Jordan**	122518.9	1.069571	0.735	ASPR
**Kazakhstan**	47835.11	1.428979	0.8	ASPR
**Kenya**	166075.6	0.659176	0.59	ASPR
**Kiribati**	1209.639	1.302427	0.612	ASPR

**Kuwait**	77192.1	0.963515	0.803	ASPR
**Kyrgyzstan**	12848.81	0.502061	0.672	ASPR
**Latvia**	2189.835	1.223068	0.847	ASPR
**Lebanon**	63948.71	1.04065	0.757	ASPR
**Lesotho**	9746.537	1.523626	0.52	ASPR
**Liberia**	15761.81	1.633838	0.435	ASPR
**Libya**	89848.84	0.565122	0.706	ASPR
**Lithuania**	3130.335	1.48065	0.858	ASPR
**Luxembourg**	10920.52	0.954835	0.904	ASPR
**Madagascar**	63585.53	0.593492	0.519	ASPR
**Malawi**	61454.81	0.594797	0.477	ASPR
**Malaysia**	698779.2	2.22043	0.802	ASPR
**Maldives**	7392.341	4.00118	0.717	ASPR
**Mali**	50085.26	1.514637	0.427	ASPR
**Malta**	6826.319	1.117398	0.878	ASPR
**Mauritania**	15340.89	0.918386	0.52	ASPR
**Mauritius**	24348.42	1.875216	0.79	ASPR
**Mexico**	2399259	-0.59549	0.774	ASPR
**Mongolia**	7953.058	1.434564	0.741	ASPR
**Montenegro**	630.8039	1.126156	0.814	ASPR
**Morocco**	394014.2	1.182671	0.667	ASPR
**Mozambique**	88920.5	1.536826	0.437	ASPR
**Namibia**	10522.63	1.031876	0.647	ASPR
**Nepal**	110276.9	2.174409	0.574	ASPR
**Netherlands**	227883.6	0.802693	0.931	ASPR
**Nicaragua**	74674.01	1.281428	0.658	ASPR
**Niger**	42719.64	1.021964	0.354	ASPR
**Nigeria**	691926.9	0.927434	0.532	ASPR
**North Macedonia**	2025.762	1.353276	0.757	ASPR
**Norway**	85017.14	0.294857	0.953	ASPR
**Oman**	47368.77	2.105717	0.821	ASPR
**Pakistan**	770343.5	0.517634	0.562	ASPR
**Palau**	224.88	1.244837	0.798	ASPR
**Palestine**	48156.49	0.870543	0.686	ASPR
**Panama**	45912.39	1.568552	0.789	ASPR
**Papua New Guinea**	75657.24	1.206293	0.544	ASPR
**Paraguay**	21153.8	1.955193	0.702	ASPR
**Peru**	549396.3	1.379303	0.75	ASPR
**Philippines**	1491859	2.3568	0.699	ASPR
**Poland**	46198.78	0.1671	0.865	ASPR
**Portugal**	152351.8	0.907269	0.847	ASPR
**Qatar**	25366.3	0.677955	0.856	ASPR
**Romania**	17462.94	1.405552	0.811	ASPR
**Rwanda**	42093.01	0.926376	0.524	ASPR
**Saint Kitts and Nevis**	539.211	1.025313	0.778	ASPR
**Saint Lucia**	1301.783	0.818816	0.747	ASPR
**Saint Vincent and the**	807.5976	1.61816	0.723	ASPR

Grenadines				
Samoa	2408.805	0.976725	0.713	ASPR
Sao Tome and Principe	699.645	1.210395	0.589	ASPR
Saudi Arabia	525370.2	1.434636	0.853	ASPR
Senegal	45370.45	0.588027	0.505	ASPR
Serbia	7604.718	1.273439	0.787	ASPR
Seychelles	1714.978	1.162537	0.797	ASPR
Sierra Leone	26872.55	1.77723	0.419	ASPR
Singapore	114388.1	1.892462	0.932	ASPR
Slovakia	5303.462	1.185796	0.855	ASPR
Slovenia	1940.492	1.357238	0.896	ASPR
Solomon Islands	5557.224	1.475163	0.546	ASPR
South Africa	342147.7	1.111306	0.699	ASPR
South Sudan	22416.1	0.205528	0.388	ASPR
Spain	675471.9	0.830596	0.891	ASPR
Sri Lanka	352838.7	2.234931	0.77	ASPR
Sudan	384497.3	2.232571	0.502	ASPR
Suriname	4397.746	1.133739	0.72	ASPR
Sweden	108772.5	0.396415	0.933	ASPR
Switzerland	120279.1	0.492402	0.944	ASPR
Tajikistan	15997.59	1.01305	0.65	ASPR
Thailand	1262714	2.632651	0.755	ASPR
Timor-Leste	12327.67	2.227969	0.625	ASPR
Togo	24157.93	1.317298	0.503	ASPR
Tonga	1412.931	0.839334	0.726	ASPR
Trinidad and Tobago	11192.53	1.565966	0.784	ASPR
Tunisia	129759.8	1.457495	0.735	ASPR
Turkey	898977.9	1.526226	0.791	ASPR
Turkmenistan	11959.35	1.439765	0.706	ASPR
Uganda	116702.2	0.852681	0.516	ASPR
Ukraine	49195.92	0.896231	0.751	ASPR
United Arab Emirates	84560.18	1.050423	0.863	ASPR
United Kingdom	1142498	0.578475	0.922	ASPR
Uruguay	31842.89	1.944867	0.804	ASPR
Uzbekistan	86003.35	1.294739	0.71	ASPR
Vanuatu	2760.523	1.363822	0.603	ASPR
Yemen	194542.3	1.193936	0.452	ASPR
Zambia	73826.06	0.557099	0.588	ASPR
Zimbabwe	58738.17	-0.12733	0.535	ASPR

S5 Table. ARIMA prediction of ASPR and ASIR 2020 to 2042.

Years	ASPR	LOW80	HIGH80	LOW95	HIGH95	ASIR	LOW80	HIGH80	LOW95	HIGH95
**2020**	1715.233	1713.514	1716.952	1712.604	1717.862	60.94745	60.87619	61.01871	60.83846	61.05644
**2021**	1755.741	1749.798	1761.684	1746.652	1764.83	62.20972	61.96493	62.45451	61.83535	62.5841
**2022**	1797.206	1783.474	1810.938	1776.205	1818.207	63.50634	62.99864	64.01404	62.72988	64.28279
**2023**	1840.193	1815.497	1864.89	1802.423	1877.963	64.88238	64.04095	65.72382	63.59552	66.16924
**2024**	1886.258	1848.197	1924.319	1828.049	1944.467	66.3758	65.14412	67.60747	64.49211	68.25948
**2025**	1936.205	1882.747	1989.662	1854.449	2017.961	68.0082	66.32507	69.69132	65.43408	70.58231
**2026**	1989.755	1918.708	2060.803	1881.098	2098.413	69.76278	67.55549	71.97007	66.38702	73.13854
**2027**	2046.27	1955.056	2137.484	1906.771	2185.769	71.62349	68.80671	74.44026	67.3156	75.93137
**2028**	2105.442	1991.193	2219.69	1930.714	2280.169	73.57885	70.06163	77.09606	68.19973	78.95797
**2029**	2167.403	2027.156	2307.649	1952.914	2381.891	75.63487	71.32466	79.94508	69.04298	82.22676
**2030**	2232.416	2063.212	2401.619	1973.641	2491.19	77.79815	72.60262	82.99368	69.85228	85.74403
**2031**	2300.596	2099.461	2501.732	1992.987	2608.206	80.0745	73.89944	86.24956	70.63056	89.51844
**2032**	2371.884	2135.759	2608.009	2010.762	2733.006	82.46189	75.20973	89.71405	71.37067	93.55311
**2033**	2446.171	2171.88	2720.461	2026.68	2865.661	84.9577	76.52661	93.38879	72.06347	97.85193
**2034**	2523.413	2207.673	2839.153	2040.531	3006.295	87.55909	77.84375	97.27443	72.70077	102.4174
**2035**	2603.639	2243.084	2964.194	2052.218	3155.06	90.26669	79.15895	101.3744	73.27887	107.2545
**2036**	2686.893	2278.091	3095.694	2061.684	3312.101	93.08148	80.47091	105.6921	73.79528	112.3677
**2037**	2773.19	2312.64	3233.739	2068.84	3477.54	96.00483	81.77858	110.2311	74.24765	117.762
**2038**	2862.518	2346.644	3378.392	2073.557	3651.479	99.03657	83.07921	114.9939	74.63189	123.4413
**2039**	2954.859	2380.005	3529.714	2075.695	3834.024	102.1764	84.3697	119.983	74.94343	129.4093
**2040**	3050.208	2412.639	3687.777	2075.131	4025.285	105.4235	85.64679	125.2003	75.17762	135.6694
**2041**	3148.57	2444.482	3852.657	2071.761	4225.378	108.7781	86.90799	130.6483	75.33063	142.2256
**2042**	3249.951	2475.471	4024.432	2065.486	4434.417	112.2403	88.15105	136.3295	75.39898	149.0816

S6 Table. BAPC prediction of ASPR and prevalence rate of age groups from 2020 to 2042.

Years	ASPR (per 100000)	Prevalence rate [age groups (per 100000)]								
		10 to 14	15 to 19	20 to 24	25 to 29	30 to 34	35 to 39	40 to 44	45 to 49	50 to 54
**2020**	2731.775647	540.1698	2494.999	3472.405	3557.97	3591.114	3581.547	3595.98	3066.208	798.3942
**2021**	2774.762763	547.2052	2533.175	3533.144	3627.968	3639.507	3637.263	3642.312	3117.295	808.3217
**2022**	2818.327697	554.4133	2568.729	3591.508	3695.428	3694.168	3701.608	3682.028	3174.863	817.2502
**2023**	2863.170333	561.8225	2602.84	3649.316	3761.951	3756.967	3771.028	3718.233	3238.122	826.8791
**2024**	2909.361626	569.4626	2636.828	3709.121	3830.573	3826.349	3836.903	3760.555	3300.786	838.6682
**2025**	2955.802849	577.3648	2671.604	3771.007	3901.95	3902.102	3894.818	3810.679	3354.481	851.1363
**2026**	3001.588996	585.5619	2707.3	3830.015	3971.56	3980.23	3948.652	3871.281	3398.861	865.6108
**2027**	3047.369736	594.0886	2744.057	3885.347	4038.808	4055.889	4009.586	3941.367	3437.32	881.9541
**2028**	3093.902212	602.9815	2782.023	3938.783	4105.75	4130.847	4079.67	4017.177	3472.756	899.9508
**2029**	3141.774159	612.2794	2821.354	3992.334	4175.279	4208.461	4157.247	4089.552	3514.174	917.8607
**2030**	3190.680941	622.0232	2862.216	4047.404	4247.526	4289.49	4242.133	4153.809	3563.183	933.3598
**2031**	3239.445647	632.2567	2904.784	4104.21	4316.91	4368.97	4329.998	4214.072	3622.297	946.3481
**2032**	3288.335481	643.0263	2949.24	4162.979	4382.521	4446.245	4415.58	4282.278	3690.613	957.7667
**2033**	3337.976184	654.3816	2995.78	4223.953	4446.364	4523.586	4500.815	4360.645	3764.638	968.4222
**2034**	3388.63144	666.3758	3044.61	4287.385	4510.724	4604.212	4589.39	4447.452	3835.815	980.83
**2035**	3440.134492	679.0655	3095.952	4353.547	4577.22	4688.313	4682.18	4542.557	3899.771	995.45
**2036**	3491.130216	692.5121	3150.04	4422.723	4646.117	4769.731	4773.775	4641.349	3960.362	1012.992
**2037**	3541.873435	706.7812	3207.125	4495.217	4717.693	4847.452	4863.462	4738.201	4028.812	1033.213
**2038**	3593.151666	721.944	3267.475	4571.352	4792.246	4923.691	4953.735	4835.203	4107.247	1055.147
**2039**	3645.59851	738.0774	3331.38	4651.472	4870.089	5000.994	5048.153	4936.35	4194.101	1076.404
**2040**	3699.325204	755.2648	3399.149	4735.944	4951.558	5081.197	5146.985	5042.644	4289.309	1095.762
**2041**	3752.777665	773.5967	3471.118	4825.163	5037.007	5164.623	5243.479	5148.276	4388.545	1114.299
**2042**	3805.53378	793.1719	3547.649	4919.55	5126.817	5251.612	5336.513	5252.479	4486.511	1135.175

S7 Table. BAPC prediction of ASIR and incidence rate of age groups from 1990 to 2042.

Years	ASIR (per 100000)	Incidence rate [age groups (per 100000)]								
		10 to 14	15 to 19	20 to 24	25 to 29	30 to 34	35 to 39	40 to 44	45 to 49	50 to 54
**2020**	94.74408	318.381	351.8209	16.85227	9.177711	4.973181	3.602536	2.332836	1.216511	0.379931
**2021**	95.67953	321.5696	355.4651	17.06616	9.258901	4.978786	3.587149	2.315586	1.208197	0.377234
**2022**	96.61127	324.8367	359.0481	17.27976	9.349082	4.989815	3.576769	2.296739	1.200591	0.374274
**2023**	97.54913	328.196	362.66	17.4857	9.444893	5.005254	3.568072	2.27559	1.192548	0.371194
**2024**	98.49722	331.662	366.3552	17.68091	9.54589	5.024667	3.557945	2.251973	1.181971	0.367999
**2025**	99.45513	335.2493	370.1559	17.86562	9.653162	5.049682	3.546505	2.227141	1.166945	0.364454
**2026**	100.42305	338.9736	374.0782	18.04493	9.765937	5.081885	3.536812	2.203642	1.147242	0.359982
**2027**	101.40127	342.8508	378.1377	18.22464	9.882859	5.121552	3.53278	2.183469	1.124247	0.353685
**2028**	102.38998	346.8978	382.3508	18.41053	10.00188	5.169029	3.536575	2.167301	1.100086	0.344832
**2029**	103.38906	351.1323	386.7348	18.6064	10.12076	5.224423	3.548945	2.154727	1.076993	0.333173
**2030**	104.39827	355.5729	391.3083	18.81388	10.23765	5.287165	3.569975	2.145534	1.056558	0.319047
**2031**	105.41774	360.2393	396.0908	19.03331	10.35383	5.355461	3.599091	2.140462	1.039749	0.303265
**2032**	106.44765	365.1521	401.1034	19.26481	10.47187	5.427681	3.635203	2.140846	1.026801	0.286756
**2033**	107.48815	370.3333	406.368	19.5086	10.59477	5.502227	3.677833	2.147251	1.017333	0.270393
**2034**	108.53922	375.8065	411.908	19.76506	10.72465	5.577627	3.726706	2.159682	1.010619	0.254965
**2035**	109.60074	381.5964	417.7483	20.0348	10.86254	5.652763	3.781274	2.17797	1.006252	0.241021
**2036**	110.67281	387.7297	423.9151	20.31862	11.00883	5.728238	3.840203	2.201661	1.004465	0.228903
**2037**	111.75554	394.2348	430.4366	20.6175	11.16386	5.805503	3.902334	2.23005	1.005895	0.218706
**2038**	112.84906	401.1425	437.3427	20.9326	11.328	5.886321	3.966548	2.262862	1.010802	0.210303
**2039**	113.95337	408.4855	444.6657	21.26523	11.50166	5.971972	4.031834	2.299968	1.019176	0.203428
**2040**	115.06846	416.2994	452.4399	21.61681	11.68532	6.063166	4.097444	2.341069	1.030921	0.197917
**2041**	116.19444	424.6225	460.7026	21.98893	11.87956	6.160232	4.163877	2.385351	1.045795	0.193826
**2042**	117.33142	433.4965	469.4937	22.3833	12.08505	6.263483	4.232238	2.432115	1.063433	0.191358
