# Supplementary material for: Global burden and epidemiological prediction of polycystic ovary syndrome from 1990 to 2019: A systematic analysis from the Global Burden of Disease Study 2019
Source: PLoS One. 2024 Jul 18;19(7):e0306991. doi: 10.1371/journal.pone.0306991 (PMC11257291; doi:10.1371/journal.pone.0306991)
Supplement: Attachment — Submitted filename: Response to Reviewers.docx [file pone.0306991.s002.docx]

**Response to Reviewers**

**Reviewer #1:**

**The manuscript is well-organized, presenting the research findings in a clear and understandable manner. The language used throughout is articulate and clear of any discernible spelling or grammatical errors. The conclusion drawn by the authors is both reasonable and well-supported by the comprehensive data provided.**

**Response:** Thank you for recognizing our work, this is greatly helpful to us.

**Reviewer #2:**

Dear authors,

Thank you for submitting your work based on the Global Burden of Disease (2019) about the assessment of global, regional and national burden of PCOS in terms of incidence, prevalence, and years lived with disability (DLYs).

PCOS is a widespread disease affecting women during their reproductive years and beyond with its metabolic and oncological complications. It is an important health problem that should be addressed and concrete strategies drawn up.

I thank the authors for this article which could benefit the current efforts made globally, regionally, and nationally in order to advance PCOS management.

The manuscript is technically sound, and the methods permitted a thorough statistical analysis of the data.

However, I have few observations:

1. **In the method section the correlation coefficient used was not mentioned.**

**Response:** Thank you for pointing out the issue. We have added the calculation method for the correlation coefficient in the “Cluster analysis and correlation analysis” section.

**2. The manuscript could benefit from editing. It must be clearer, with no ambiguity, which was the case for many sentences in the result sections.**

**148: There was an increase of 32 million PCOS patients worldwide…**

**149: The incidence of PCOS in one year increased from 1.4 million in 1990 to 2.1 million in 2019.**

**152: the region which had the highest incidence in 1990 ……..was the western pacific…..**

**154: this sentence should be rephrased so that we know that EAPC of incidence estimates of the Americas region was only one that decreased.**

**160:** **ASR were the highest in Region of the Americas in 1990 and 2019, while the EAPC estimates were highest in South-East Asia Region.**

**Response:** Thank you very much for pointing out the grammatical issues. This has made the manuscript more fluent, and we have made the corresponding corrections as per your suggestions.

**3. The legends in all the figures were illegible, so it was impossible to read them.**

**Response:** We have redrawn all the images with fonts that were too small or potentially non-standard and uploaded high-resolution versions.

Thank you again for pointing out these issues; this has greatly improved the readability of our article.

**Reviewer #3:**

**This paper is, somehow, an interesting excercise of probabilistic (Bayesian) approaches to a previously collected data (from the Global Health data Exchange) . It is, as a hole, a very neat metodological excercise. From the clinical point of view, as it should be expected, it does not add any important data.**

**Methodologically, it is a very well designed analysis and allows to withdraw some info which, considering the heterogenicity of the sample and the variety of criteria involved, renders results that should be taken precauciously.**

**From the stylistic point of view, there are several abbreviations in the abstract that are totally inintelligible unless you read the entire paper (ASIR, HDI, ASIR and ASPIR). Those should be, either, properly adressed or retired.**

**Response:** Thank you for pointing out the errors. We have added the full names for PCOS, ASIR, ASPR, and HDI in the abstract section.
